# Beta-caryophyllene as an antioxidant, anti-inflammatory and re-epithelialization activities in a rat skin wound excision model

**DOI:** 10.1155/2022/9004014

**Published:** 2022-02-03

**Authors:** Lucas Fernando Sérgio Gushiken, Fernando Pereira Beserra, Maria Fernanda Hussni, Murilo Tireli Gonzaga, Victor Pena Ribeiro, Patrícia Fernanda de Souza, Jacqueline Costa Lima Campos, Taís Nader Chrysostomo Massaro, Carlos Alberto Hussni, Regina Kiomi Takahira, Priscyla Daniely Marcato, Jairo Kenupp Bastos, Cláudia Helena Pellizzon

**Affiliations:** ^1^São Paulo State University-UNESP, Institute of Biosciences of Botucatu-IBB, São Paulo, Brazil; ^2^São Paulo State University-UNESP, School of Veterinary Medicine and Zootechnics-FMVZ, São Paulo, Brazil; ^3^University of São Paulo-USP, School of Pharmaceutical Science of Ribeirão Preto-FCFRP, São Paulo, Brazil

## Abstract

The skin is a critical organ for the maintenance of the integrity and protection of the organism. When a wound occurs, a sequence of healing mechanisms is triggered to reconstruct the wounded area. *β*-caryophyllene is a sesquiterpene in *Copaifera langsdorffii* oleoresin with antioxidant and anti-inflammatory potential. On the basis of previous studies with *C. langsdorffii*, *β*-caryophyllene was selected to evaluate its wound healing potential and pharmacological mechanisms. The excision wound model was used with male *Wistar* rats and macroscopic, histological, immunohistochemical and biochemical analyses were performed with skin samples, comparing the *β*-caryophyllene-treated group with reference drugs. The results showed macroscopic retraction of the wounds treated with *β*-caryophyllene. Biochemical assays revealed the antioxidant and anti-inflammatory mechanisms of the *β*-caryophyllene-treated group with increasing levels of IL-10 and GPx and decreasing levels of pro-inflammatory molecules, including TNF-*α*, IFN-*γ*, IL-1*β* and IL-6. After *β*-caryophyllene treatment, immunohistochemical assays showed enhanced re-epithelialization, through the increase in laminin-*γ*2 and desmoglein-3 immunolabeling. *β*-caryophyllene also act in the remodeling mechanism, increasing the collagen content in the Masson's trichrome staining. These findings indicated the wound-healing potential of *β*-caryophyllene topical formulation in rat skin wounds, mediated by antioxidant, anti-inflammatory and re-epithelialization mechanisms.

## 1. Introduction

Skin wounds are major health problems that affect millions of people every year, causing physical and psychological deficiencies when not treated correctly [1]. The comorbidities associated with unhealed wounds increase every year, and the costs of healing treatments have reached billions of dollars worldwide [2]. The wound-healing process involves overlapping and interdependent mechanisms (inflammation, epithelialization, angiogenesis, wound retraction and matrix remodeling) to reconstruct the skin [3]. When there is an imbalance among these mechanisms, the healing process enters a pathologic state, resulting in errors of healing, such as hypertrophic scars and unhealed wounds [4]. To prevent pathologic mechanisms of wound healing, there are several treatments on the market used to promote cutaneous healing by acting as antimicrobial or anti-inflammatory agents, improving tissue debridement, epithelialization and/or remodeling mechanisms. However, the existing treatments may not be efficient in treating cutaneous wounds depending on the type, extension and location of the injury [5]. Therefore, studies have focused on discovering alternative drugs that accelerate skin wound healing without scarring [6].

*β*-caryophyllene (trans-(1,9)-8-methylene-4,11,11-trimethylbicycloundec-4-ene) is a natural bicyclical sesquiterpene found in several plants and essential oils, including *C. langsdorffii* (Leguminosae) oil [7]. *β*-caryophyllene is a volatile compound that is poorly soluble in water and has high pharmaceutical potential due to its analgesic, antioxidant, antimicrobial and anti-inflammatory activities [8–10]. Previous studies on *C. langsdorffii* have suggested the healing potential of *C. langsdorffii* oil resin in a 10% formulation [11]. Therefore, our group tested a 1% *β*-caryophyllene emulgel formulation to analyze the wound-healing potential and the mechanisms of action of this new formulation in a rat excision wound model.

## 2. Materials and Methods

### 2.1. Chemicals and reagents

The reference drugs neomycin sulfate (5 mg/g) + bacitracin zinc (250 IU/g), dexpanthenol 5% and collagenase 1.2 IU were purchased from pharmaceutical industries. SOD, CAT, MPO, GSH, GPx and silica gel 60H chromatoplates were obtained from Sigma-Aldrich Chemicals (Saint Louis, USA). ELISA kits of TNF-*α*, IFN-*γ*, IL-1*β*, IL-6 and IL-10 were bought from R&D Systems (Minneapolis, USA). Biochemical colorimetric kits for quantification of AST, ALT, *γ*-GT, urea and creatinine were purchased from Interteck-Katal (Belo Horizonte, Brazil). The primary antibodies Lam*γ*2 was purchased from Santa Cruz Biotechnology (Dallas, USA). Dsg3, Ki-67, *α*-SMA antibodies and immunohistochemistry reveal kits were purchased from Abcam (Cambridge, USA). Sepineo P600, propylene glycol and methyldibromo glutaronitrile/phenoxyethanol were obtained from Spectrum Chemical Manufacturing Corporation (New Brunswick, USA). Labrafac lipophile WL 1349 was purchased from Gattefossé (Lyon, France).

### 2.2. Extraction and isolation of *β*-caryophyllene

The extraction and isolation of *β*-caryophyllene have been previously reported by Ribeiro et al. [12] [12]. Briefly, *C. langsdorffii* oleoresin was collected in the northern and southeastern regions of Brazil, and the plant voucher was identified by Silvana Tavares Rodrigues at the Herbarium from EMBRAPA Amazônia Oriental (SPFR 10120). One hundred milliliters of a volatile fraction of oleoresin was added to 500 mL of water and subjected to hydrodistillation for 12 hours using a Clevenger-type apparatus. Then, the volatile fraction was subjected to a spinning band distillation process to obtain fractions rich in *β*-caryophyllene. *β*-caryophyllene was purified from these fractions by classical column chromatography packed with Sigma-Aldrich 60H silica gel impregnated with AgNO_3_ with a gradient of hexane-ethyl acetate used as the mobile phase.

### 2.3. Formulation of emulgel and 1% *β*-caryophyllene emulsion

The emulgel was synthesized homogenizing Sepineo P600 (3% w/w), propylene glycol (5% w/w), Labrafac lipophile WL1349 (10% w/w), methyldibromo glutaronitrile/phenoxyethanol (0.1% w/w) and water at room temperature (±25°C) with a Polytron PT 10-35 GT (Kinematica, Switzerland) at 600 rpm. The 1% *β*-caryophyllene emulsion was synthesized homogenizing the *β*-caryophyllene (1% w/w) with the emulgel at room temperature (±25°C). The rheological behavior of the emulgel was analyzed in a Rheometer R/S plus (Brookfield) equipped with a C50-1 spindle and RHEO Software 2000 version 2.8. The sample behavior was monitored at a constant temperature (25°C) using a water bath/circulator.

### 2.4. Animals

In total, 105 male 7-week-old *Wistar* rats weighing 250 ± 20 g (Central Animal House, UNESP, Botucatu) were used in the experiments. The animals were housed individually for one week before the experiments and subjected to a temperature of 23 ± 2°C and a 12-hour dark-light cycle, and the animals had free access to food and water until the experimental procedure were initiated. This study was approved by the Ethics Committee on Animal Use at São Paulo State University under protocol 976/2017.

### 2.5. Experimental protocol of excision wound

The rats were anesthetized with intraperitoneal ketamine (100 mg/kg) and xylazine (10 mg/kg) [13]. The hair of their back was shaved and a full-thickness skin wound excision was made in the dorsum (in the subscapular area) using a 3 cm diameter punch. The wound placed in this area could not be reached by the animals, which prevented self-licking [14]. Each rat was topically treated twice per day for three different experimental periods: 3, 7 or 14 days (n =35 animals/period). After each period, the animals were euthanized, and samples of the skin wounds and blood were collected for analyses. The animals were allocated into the following seven groups (n =5/group):

- FST: wounded animal without treatment
- NeBa: wounded animal treated with neomycin 5 mg/g + bacitracin zinc 250 IU/g
- Dex: wounded animals treated with dexpanthenol 5%
- Col: wounded animals treated with collagenase 1.2 IU
- Emulgel: wounded animals treated with emulgel (vehicle)
- Car: wounded animals treated with 1% *β*-caryophyllene emulgel
- Control: animals without lesion and treatment – physiologic pattern

### 2.6. Wound contraction analysis

To measure the contraction of the wounded area, each wound was photographed using a digital camera with a scale bar on days 0, 3, 7 and 14 after the excision wound was induced (day 0). A morphometric analysis was performed through measurement of wounded areas using specific software, and the percentage of wound contraction of each rat was calculated using the following formula [11, 15]:

(1) $$\begin{array}{c} \text{Wound concentration }\left(\%\right)=\left\{\left(\text{initial wound area}–\text{analyzed area}\right)/\text{initial wound area}\right\}*100 \end{array}$$

### 2.7. Hepatic and renal toxicity

Immediately after the euthanasia of each animal, blood was collected and centrifuged for 15 minutes at 6000 rpm and 4°C. The supernatant was collected and used to determine liver and kidney toxicity using AST, ALT and *γ*-GT (IU/L) parameters as well as the concentrations of urea and creatinine (mg/dL).

### 2.8. Inflammatory mediators and antioxidant enzymes

Samples of wounded skin of each animal were collected to quantify the IFN-*γ*, IL-1*β*, IL-6, IL-10 and TNF-*α* inflammatory cytokines and the antioxidant enzymes, including SOD, CAT, GPx and GSH. Halves of each wound were cut using a scalpel, including the border and center of the lesions. The skin samples were instantly homogenized (1 : 5 m/v) in phosphate buffer (pH 7.4) and centrifuged for 15 minutes at 10000 rpm and 4°C. The supernatant of each sample was collected, and the cytokines were quantified by ELISA as described by the supplier. The results were expressed in pg/mg of protein. The following molecules involved in oxidative stress pathways were quantified through biochemical assays according to protocols: SOD (IU/mg of protein) [16], CAT (IU/mg of protein) [17], GPx (nmol NADPH/min/mg of protein) [18] and GSH (nmol/mg of protein) [19].

### 2.9. Histological parameters

The other halves of wounded skin samples from each rat were fixed with 10% buffered formaldehyde, embedded in paraffin, sectioned (5 *μ*m) and stained with HE and Masson's trichrome stain. Each section was submitted to morphometric analysis via light microscopy. With HE staining, the epidermis thickness (*μ*m), number of total cells in the epidermis and dermis (*μ*m^2^) and the quantity of blood vessels in the dermis (number of blood vessels) were analyzed. With Masson's trichrome staining, the collagen content of the dermis (*μ*m^2^) was analyzed. The border and center regions of the wounds were analyzed with five different photomicrographs of each region in the sections. AvSoft BioView Spectra software was used to perform the analysis.

### 2.10. Immunohistochemistry

Skin samples from each rat were processed routinely (fixation with 10% buffered formaldehyde, embedded in paraffin and sectioned; 5 *μ*m thickness). Immunohistochemistry was then performed with primary antibodies against laminin-*γ*2 (1 : 200 *μ*L), desmoglein-3 (1 : 200 *μ*L), Ki-67 (1 : 100 *μ*L) and *α*-SMA (1 : 400 *μ*L) according to the protocols of a specific HRP/DAB detection kit. The areas (*μ*m^2^) and positive cells (count of positive cells) for each antibody were quantified in the border and center of wounds with five different photomicrographs for each region of the sections. AvSoft BioView Spectra software was used to perform the analysis.

### 2.11. Statistical analysis

The data are expressed as the means ± standard deviation. Two-way ANOVA with Bonferroni post-test was used in the analysis of wound contraction. The inflammatory mediator, oxidative stress and toxicity data were submitted to one-way ANOVA with Tukey's post-hoc test. Histological parameters and immunohistochemical data were analyzed according to the Kruskal-Wallis test with Dunn's post-test. GraphPad Prism 5.01 software (GraphPad Software Inc., San Diego, USA) was used to perform the analyses with a significance of 5%.

## 3. Results

### 3.1. Emulgel and 1% *β*-caryophyllene emulsion stability test

The rheological behavior of emulgel and 1% *β*-caryophyllene emulsion showed a non-Newtonian characteristic, with pseudoplastic behavior (n <1). This result demonstrates that, with the increase of the shear rate, the viscosity reduces (data not shown).

### 3.2. Wound contraction analysis

**Figure 1:**
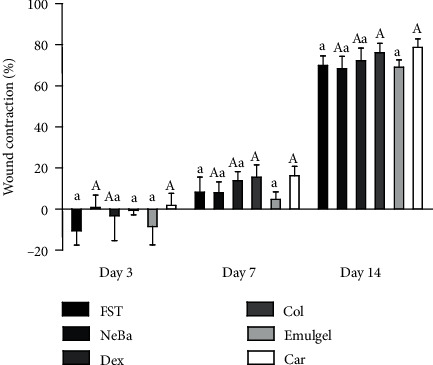
Wound contraction (%) in FST, NeBa, Dex, Col, Emulgel and Car treatments during 3, 7 and 14 days. Equal letters represents no statistical difference. Capital letters indicate statistical difference compared to groups with small letters, according to two-way ANOVA followed by Bonferroni test, with p ≤0.05 (n =5).

The contraction of the wounds was analyzed on days 3, 7 and 14 (Figure 1). The data showed macroscopic contraction of wounds and a decrease in local edema in the groups treated with NeBa and Car for three days compared to the FST and Emulgel groups. After seven and fourteen days of treatment, the Col and Car formulations showed the best results in terms of macroscopic contraction compared to the FST and emulgel formulations. Furthermore, in both periods of treatment, the rats treated with Car showed decreased fibrinous exudate compared to the other groups.

### 3.3. Hepatic and renal toxicity

To analyze the hepatic and renal toxicity of the formulation containing *β*-caryophyllene, the liver enzymes (AST, ALT and *γ*-GT) and kidney proteins (creatinine and urea) were evaluated in the blood of the rats after 14 days of treatment (the longest period of treatment). No significant differences among the values of all the treatment measurements and the normal parameters were found during the three experimental periods of treatment (Table 1).

### 3.4. Quantification of inflammatory mediators

**Figure 2:**
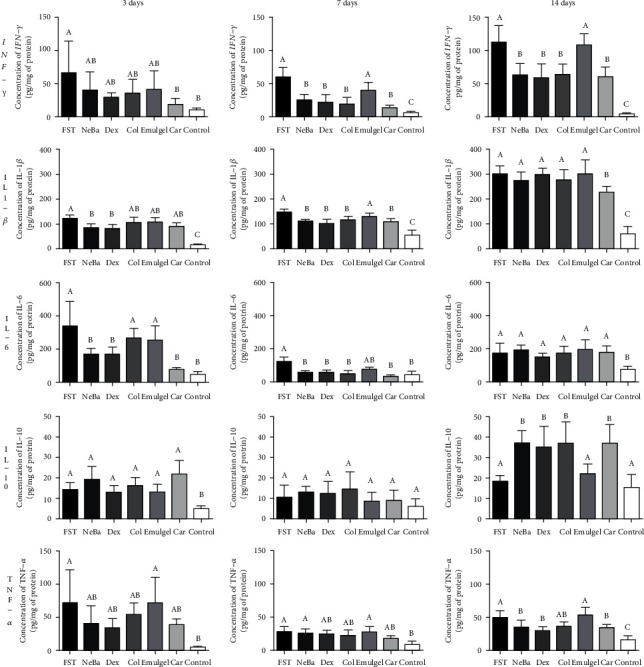
Concentrations of IFN-*γ*, IL-1*β*, IL-6, IL-10 and TNF-*α* (pg/mg protein) in cutaneous wounds at 3, 7 and 14 days. Equal letters show no statistical difference and different letters indicate statistical difference compared to the other groups, according to one-way ANOVA followed by Tukey test, with p ≤0.05 (n =5).

The concentrations of the IFN-*γ*, IL-1*β*, IL-6, IL-10 and TNF-*α* cytokines as evaluated by ELISA are shown in Figure 2. The level of IFN-*γ* was reduced in the Car group compared to the FST group after three days. In the two other treatment periods, the NeBa, Dex and Col commercial formulations, as well as the tested Car drug, decreased the levels of IFN-*γ* compared to the FST and Emulgel. After three days, a reduction in the IL-1*β* level in NeBa and Dex treated groups compared to FST group was observed. Within seven days, decreased levels of IL-1*β* were observed in the NeBa, Dex, Col and Car groups compared to the FST and Emulgel groups. In the last period of treatment, the Car group showed decreased IL-1*β* concentrations compared to all wounded groups. The IL-6 levels were decreased in the NeBa, Dex and Car groups after three and seven days of treatment compared to the FST and Emulgel groups. The levels of IL-10 were not different among the treatments on days three and seven. In the last period of treatment, an increase in IL-10 was observed in the NeBa, Dex, Col and Car treatment groups compared to the FST and Emulgel treatment groups. The TNF-*α* levels were reduced in the NeBa, Dex and Car groups compared to the FST and Emulgel groups on day fourteen, and there were no significant differences in the two other periods (Figure 2).

### 3.5. Oxidative stress analysis

**Figure 3:**
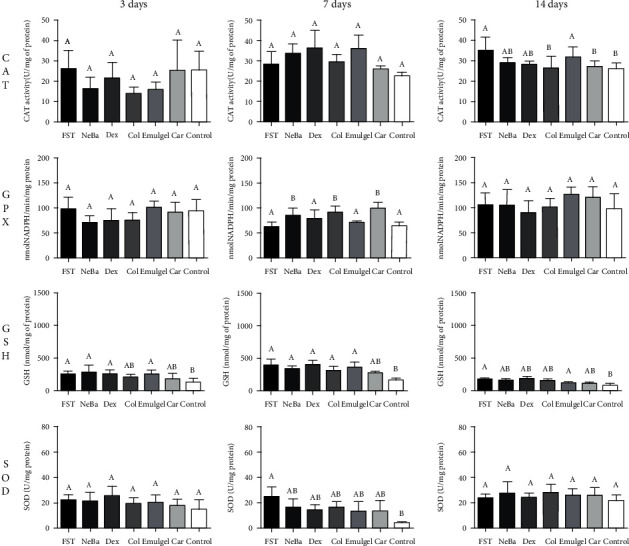
Concentrations of CAT, GPx, GSH and SOD in skin wounds at 3, 7 and 14 days. Equal letters show no statistical difference and different letters indicate statistical difference compared to the other groups, according to one-way ANOVA followed by Tukey test, with p ≤0.05 (n =5).

The CAT activity, GPx activity, SOD activity and GSH concentration are shown in Figure 3. The activity of CAT was decreased in the Col and Car groups compared to the FST and Emulgel groups on day fourteen, with the same level of Control, demonstrating the normality of the enzyme in the groups. During the first two periods of treatment, there was no significant difference in the CAT activity. The GPx activity was increased in the NeBa, Col and Car treatment groups compared to the other groups during the seven-day treatment period with no differences on days three and fourteen. The GSH concentration and SOD activity was not different among the wounded groups in any period (Figure 3).

### 3.6. Histological parameters

**Figure 4:**
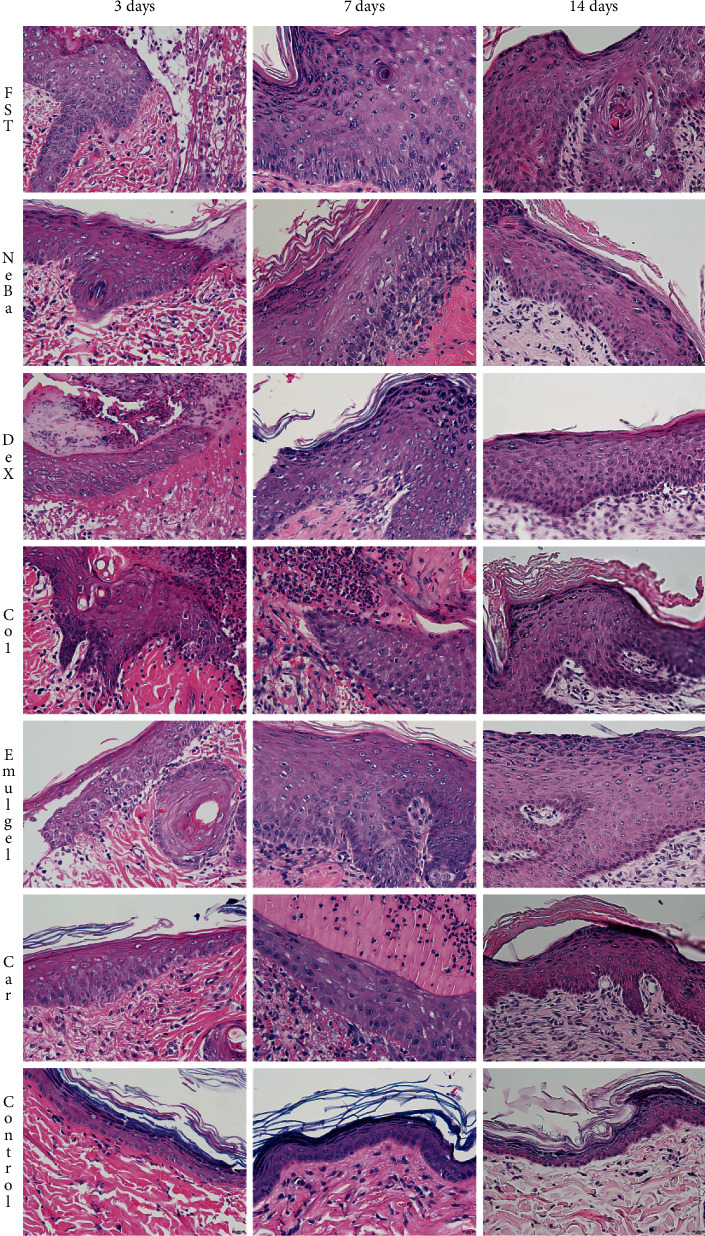
HE photomicrographs of the epidermis in FST, NeBa, Dex, Col, Emulgel, Car and Control groups during 3, 7 and 14 days.

**Figure 5:**
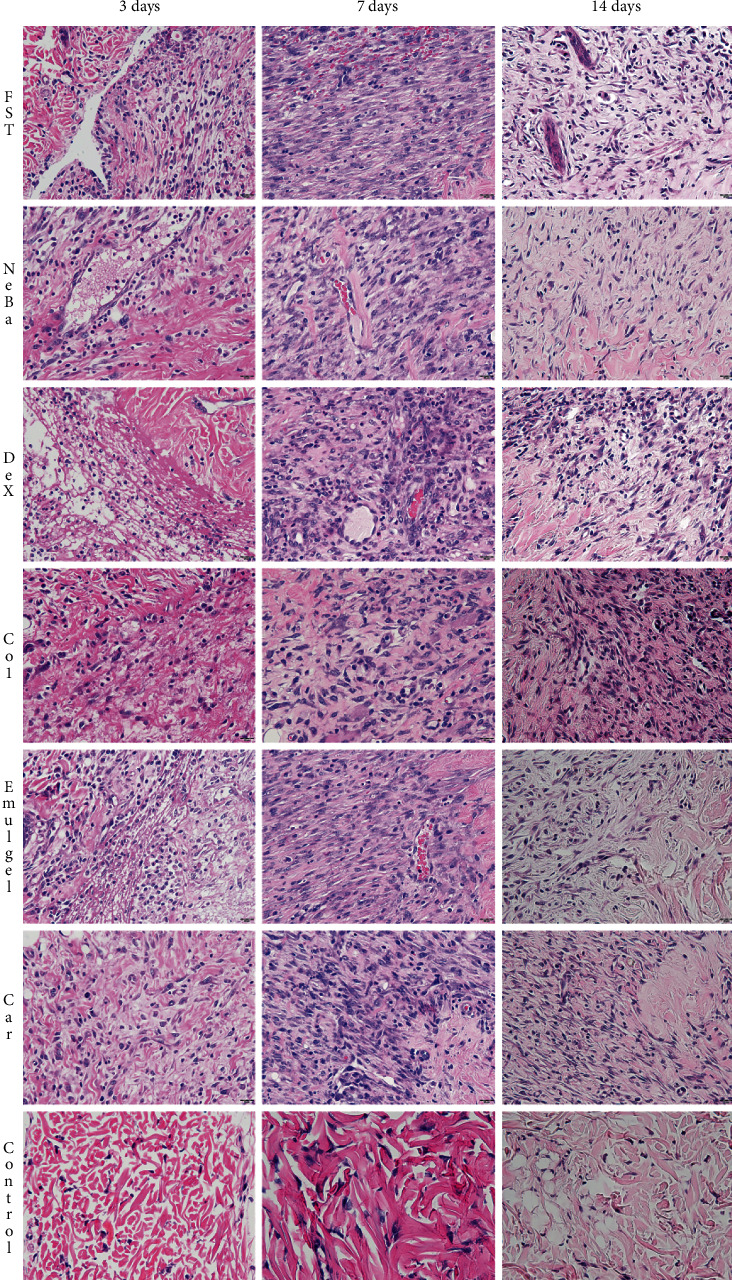
HE photomicrographs of the border of the wounds in the dermis of FST, NeBa, Dex, Col, Emulgel, Car and Control groups during 3, 7 and 14 days.

**Figure 6:**
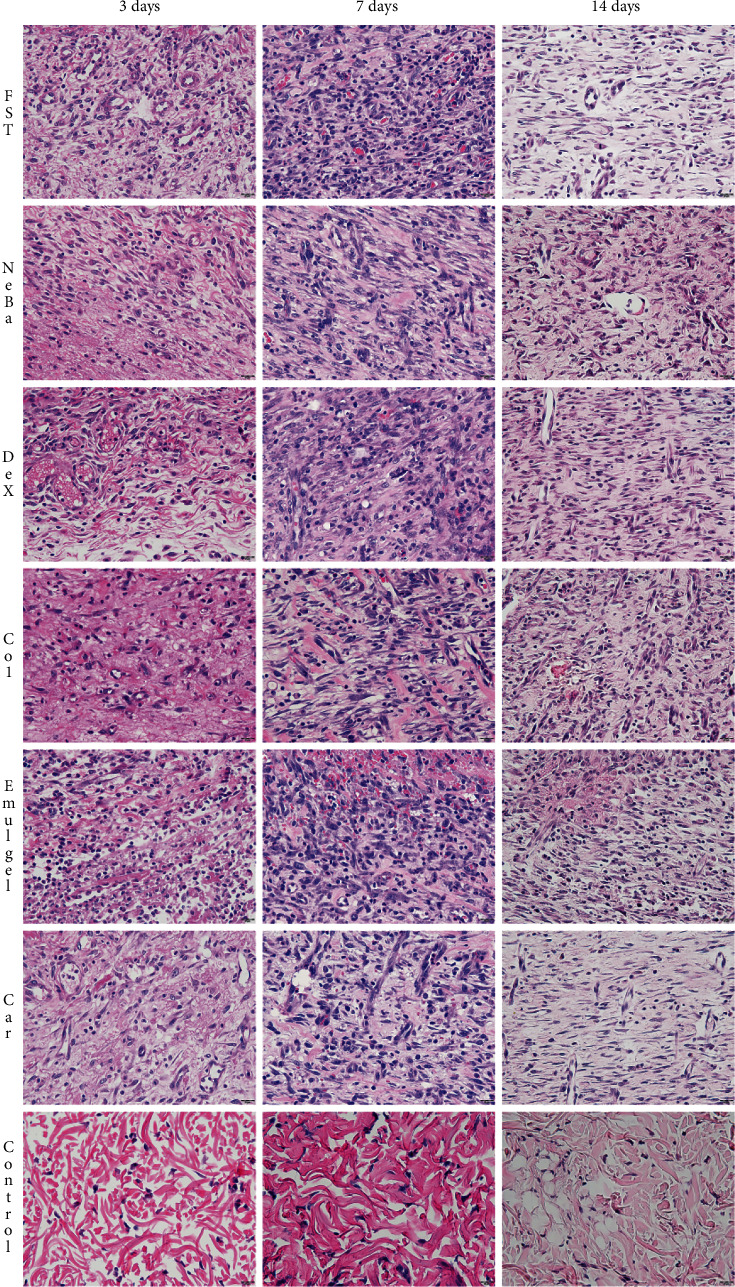
HE photomicrographs of the center of wounds in the dermis of FST, NeBa, Dex, Col, Emulgel, Car and Control groups during 3, 7 and 14 days.

**Figure 7:**
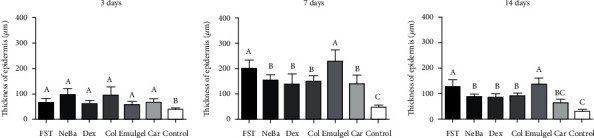
Thickness of epidermis (*μ*m) of FST, NeBa, Dex, Col, Emulgel, Car and Control groups during 3, 7 and 14 days. Equal letters show no statistical difference and different letters indicate statistical difference compared to the other groups, according to the Kruskal-Wallis test, followed by the Dunn post-test, with p ≤0.05 (n =5).

**Figure 8:**
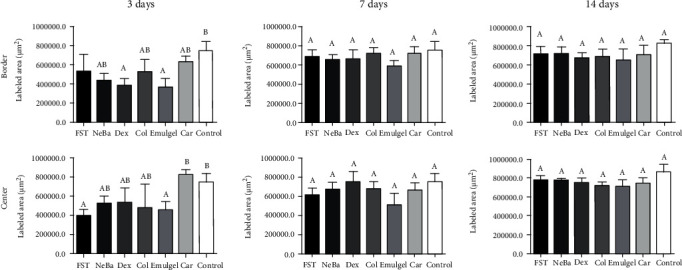
Quantification of collagen (*μ*m^2^) at the border and center of wounds of FST, NeBa, Dex, Col, Emulgel, Car and Control groups during 3, 7 and 14 days. Equal letters show no statistical difference and different letters indicate statistical difference compared to the other groups, according to the Kruskal-Wallis test, followed by the Dunn post-test, with p ≤0.05 (n =5).

**Figure 9:**
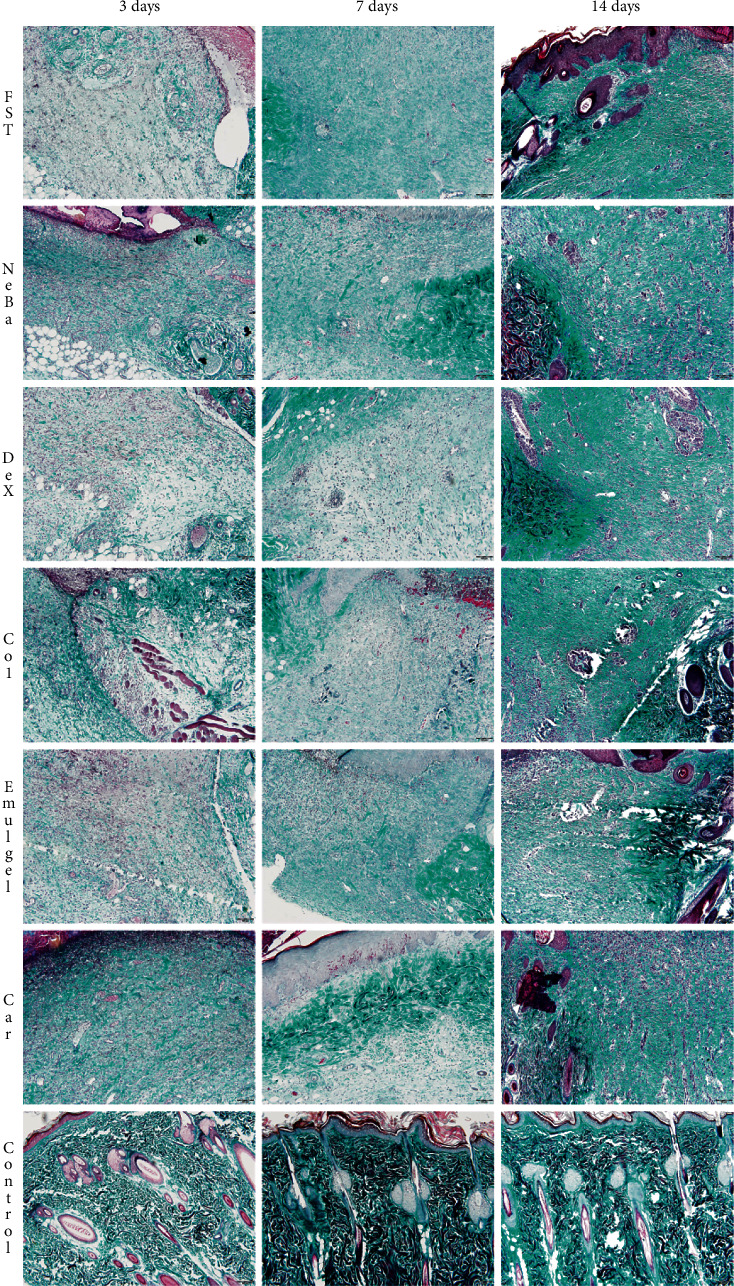
Masson's trichrome photomicrographs of the border of the wounds in the dermis of FST, NeBa, Dex, Col, Emulgel, Car and Control groups during 3, 7 and 14 days.

**Figure 10:**
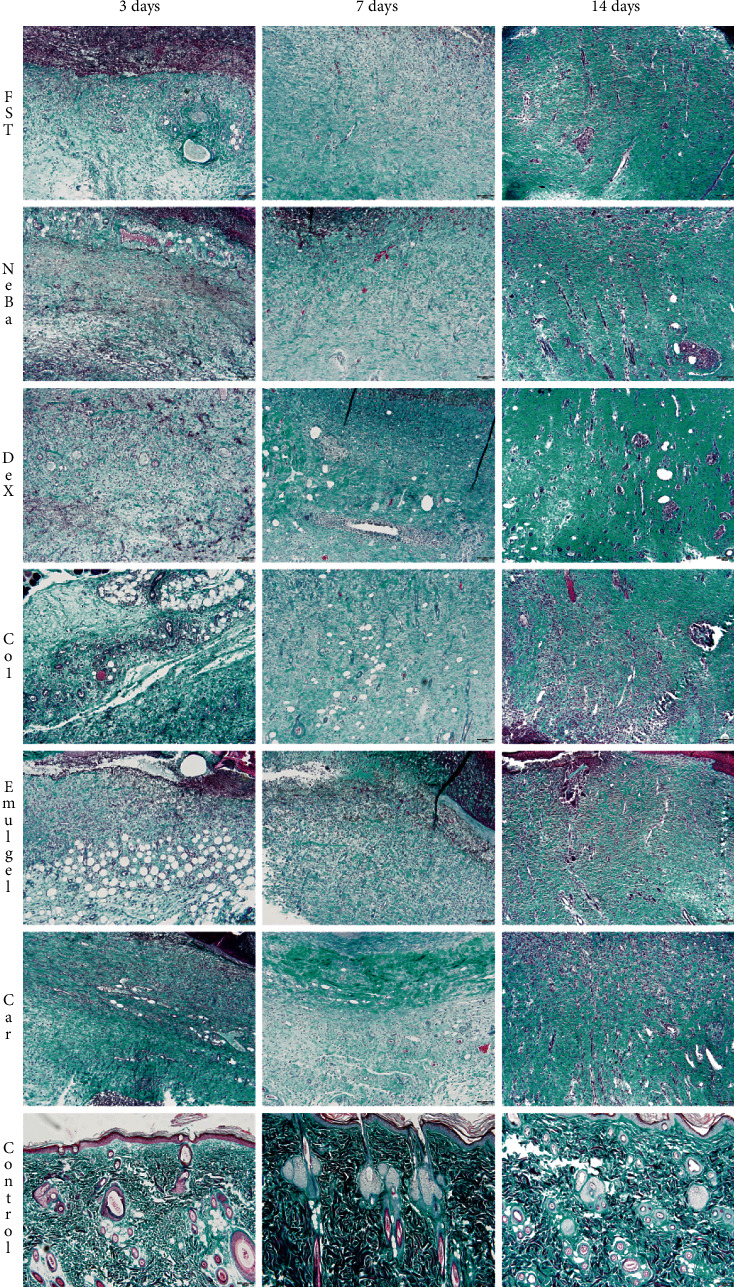
Masson's trichrome photomicrographs of the center of the wounds in the dermis of FST, NeBa, Dex, Col, Emulgel, Car and Control groups during 3, 7 and 14 days.

There was no difference in the quantification of cells from the epidermis, border of center of the dermis among the wounded groups in any period of treatment (Figures 4–6 and Supplementary materials 1). The morphometric analysis showed a reduction in the epidermis thickness of the animals treated with the NeBa, Dex, Col and Car formulations compared to that of the animals in the FST and Emulgel groups after seven and fourteen days. Moreover, the results of the Car group on day fourteen were similar to those of the Control group, resulting in normal epidermis thickness in the Car treatment (Figures 4 and 7). The quantification of blood vessels in the border and center of the wounds did not reveal a significant difference during any period of treatment (Figures 5 and 6, Supplementary materials 2). The quantification of total collagen in the border of the wounds showed no difference on days three, seven and fourteen. In the central area of wounds, there was an increase in collagen amount in the Car group compared to the FST and Emulgel on day three, with a similar concentration compared to Control. After seven and fourteen days, no differences were observed (Figures 8–10).

### 3.7. Immunohistochemistry

**Figure 11:**
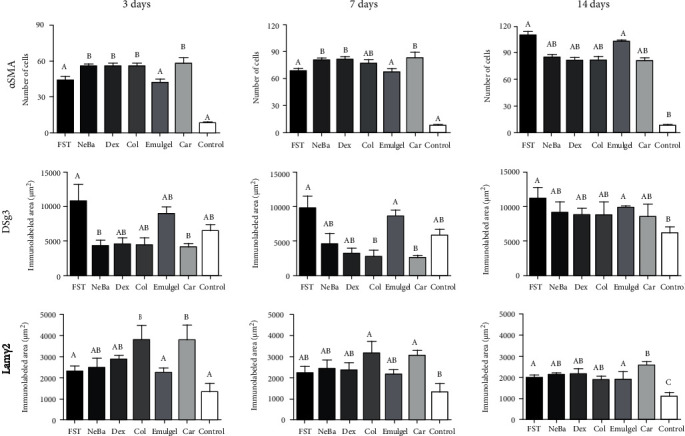
Immunolabeling of *α*-SMA, Dsg3 and Lam*γ*2 in wounds of FST, NeBa, Dex, Col, Emulgel, Car and Control groups during 3, 7 and 14 days. Equal letters show no statistical difference and different letters indicate statistical difference compared to the other groups, according to the Kruskal-Wallis test, followed by the Dunn post-test, with p ≤0.05 (n =5).

**Figure 12:**
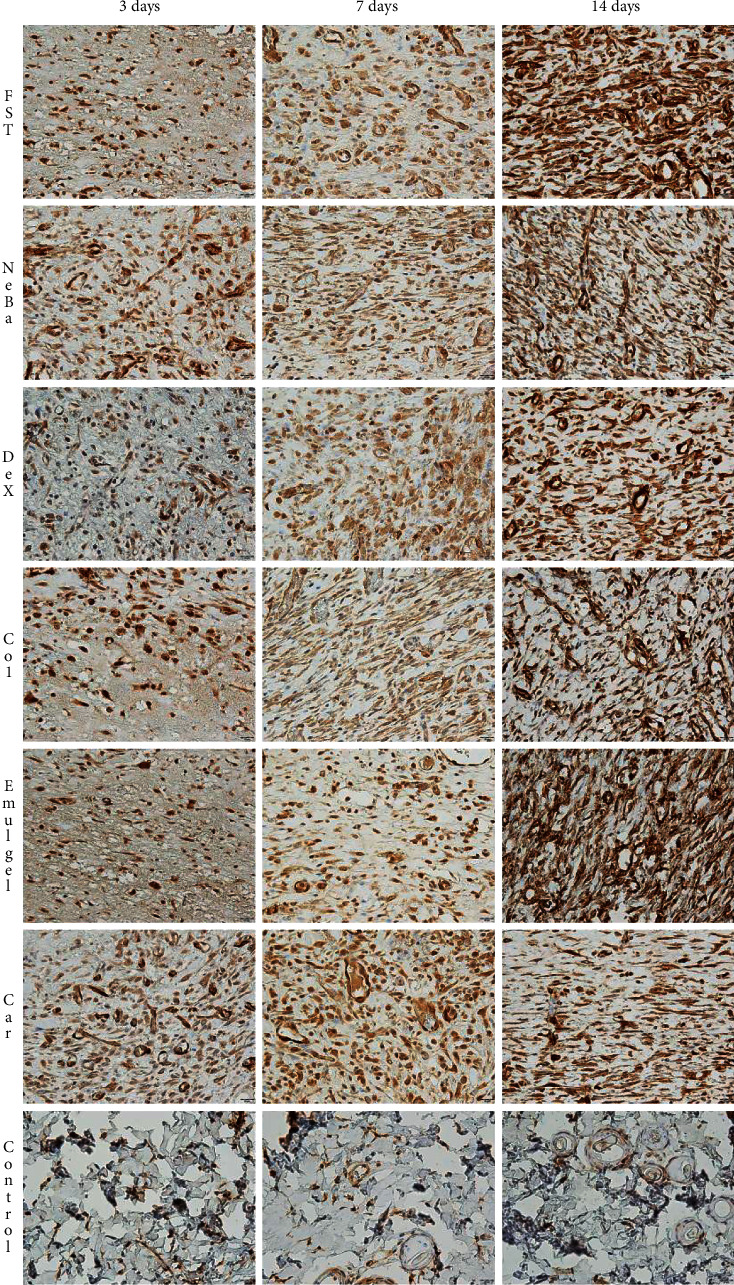
Photomicrographs of the immunolabeling of *α*-SMA in wounds of FST, NeBa, Dex, Col, Emulgel, Car and Control groups during 3, 7 and 14 days.

**Figure 13:**
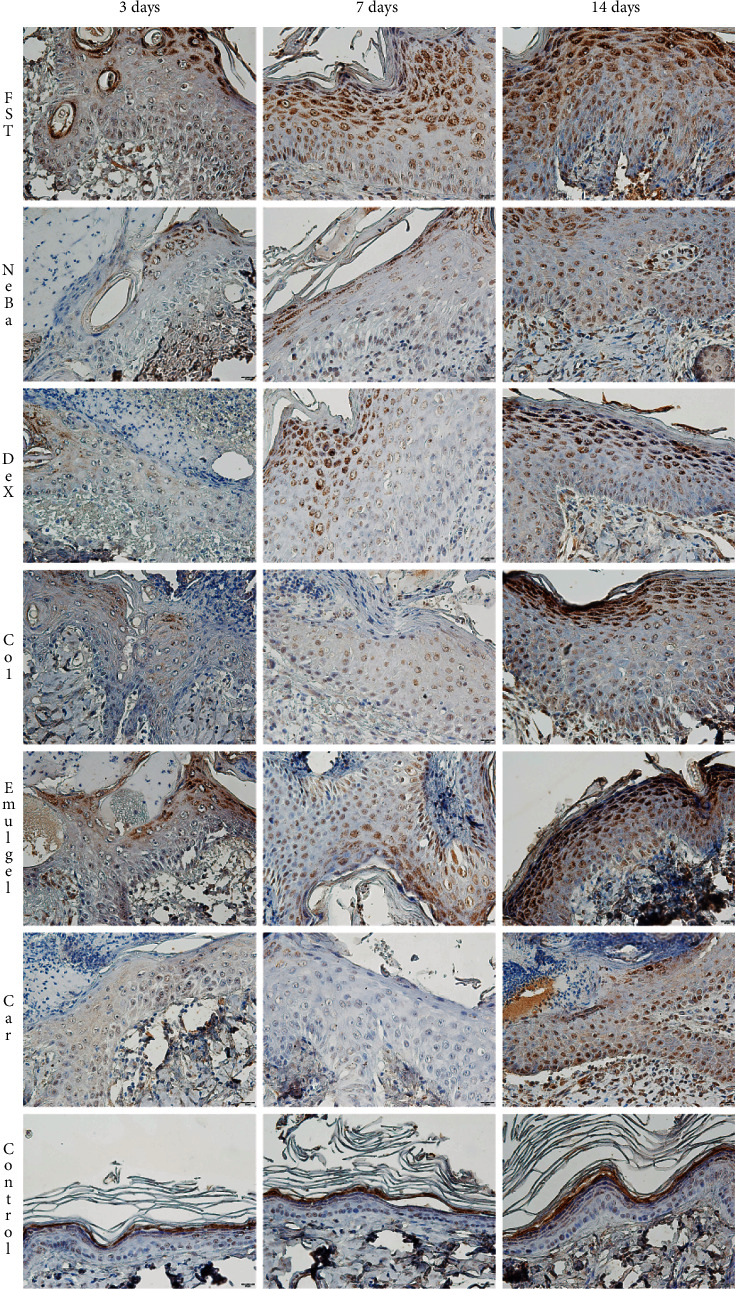
Photomicrographs of the immunolabeling of Dsg3 in wounds of FST, NeBa, Dex, Col, Emulgel, Car and Control groups during 3, 7 and 14 days.

**Figure 14:**
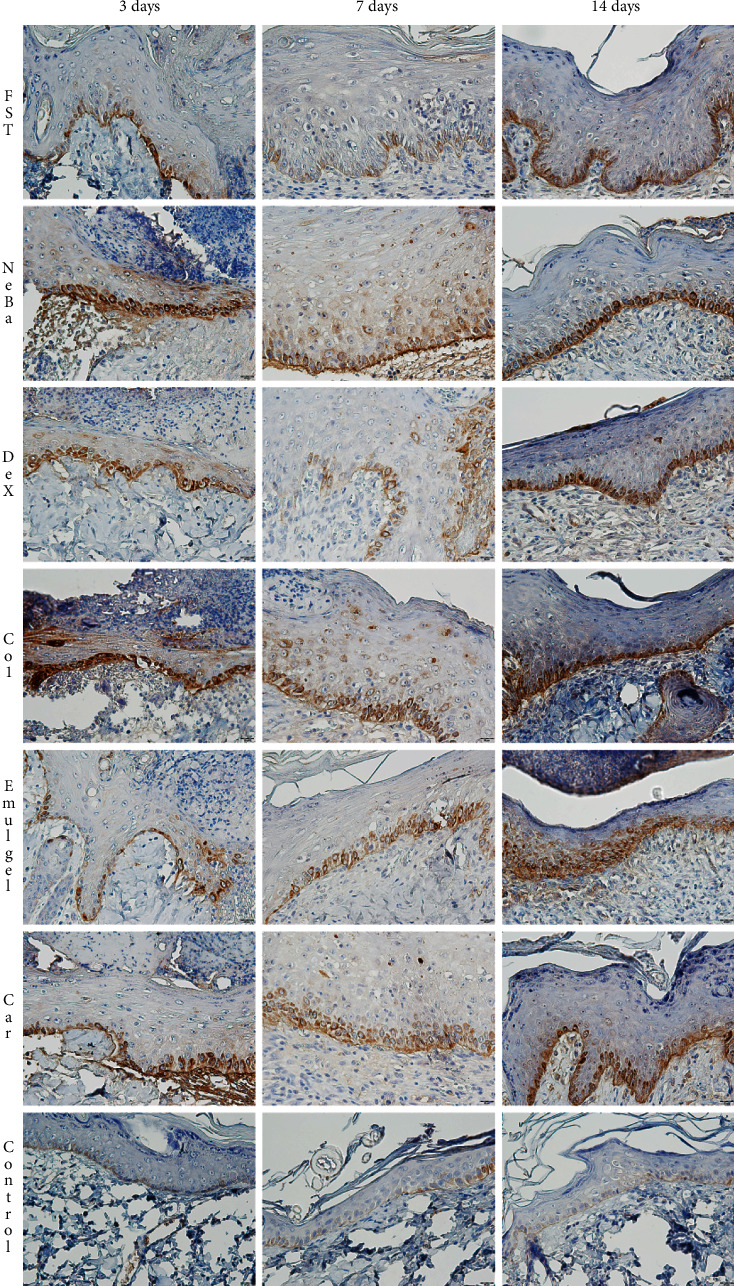
Photomicrographs of the immunolabeling of Lam*γ*2 in wounds of FST, NeBa, Dex, Col, Emulgel, Car and Control groups during 3, 7 and 14 days.

**Figure 15:**
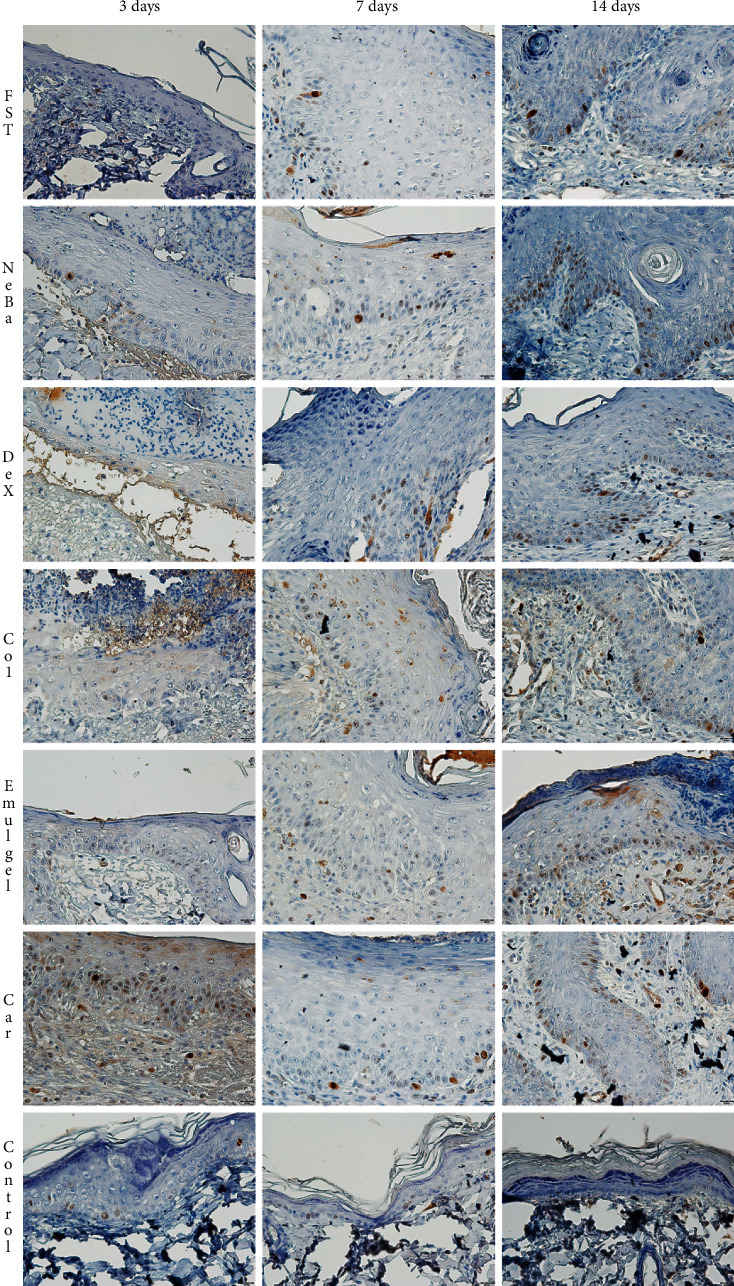
Photomicrographs of the immunolabeling of Ki-67 in the epidermis of FST, NeBa, Dex, Col, Emulgel, Car and Control groups during 3, 7 and 14 days.

**Figure 16:**
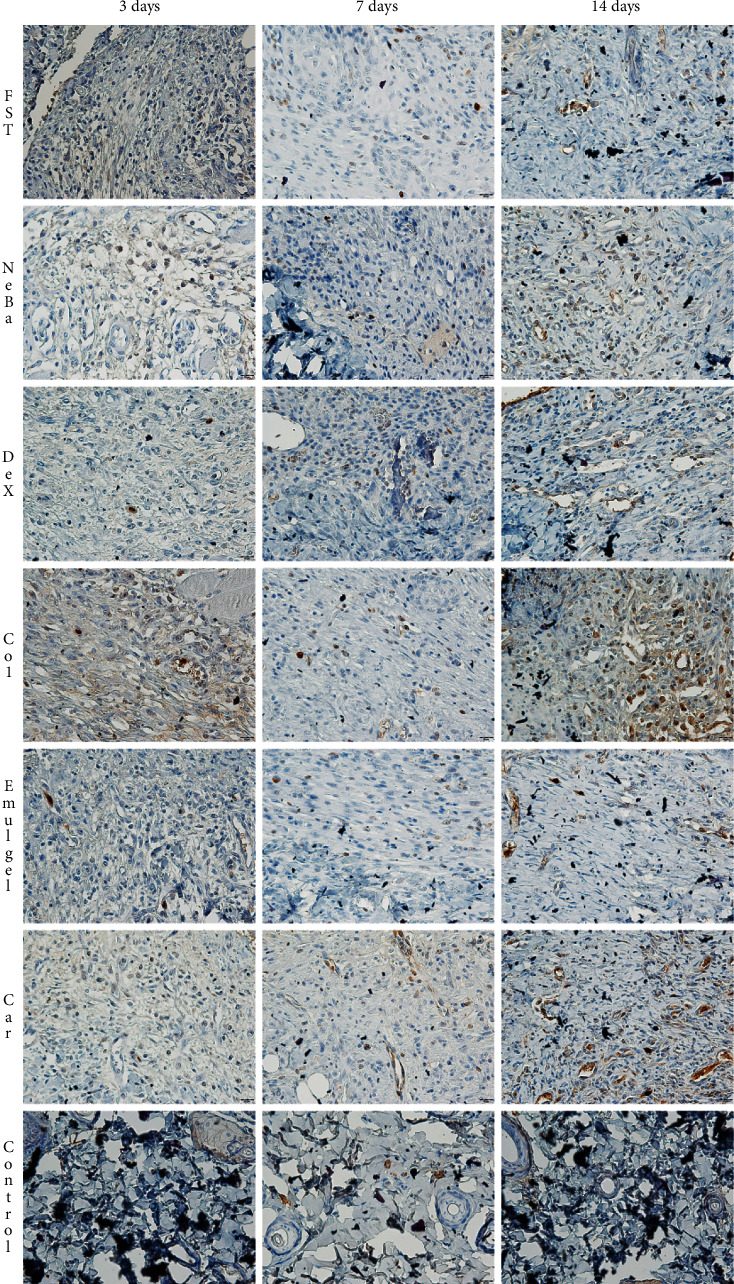
Photomicrographs of the immunolabeling of Ki-67 of the border of the wounds in the dermis of FST, NeBa, Dex, Col, Emulgel, Car and Control groups during 3, 7 and 14 days.

**Figure 17:**
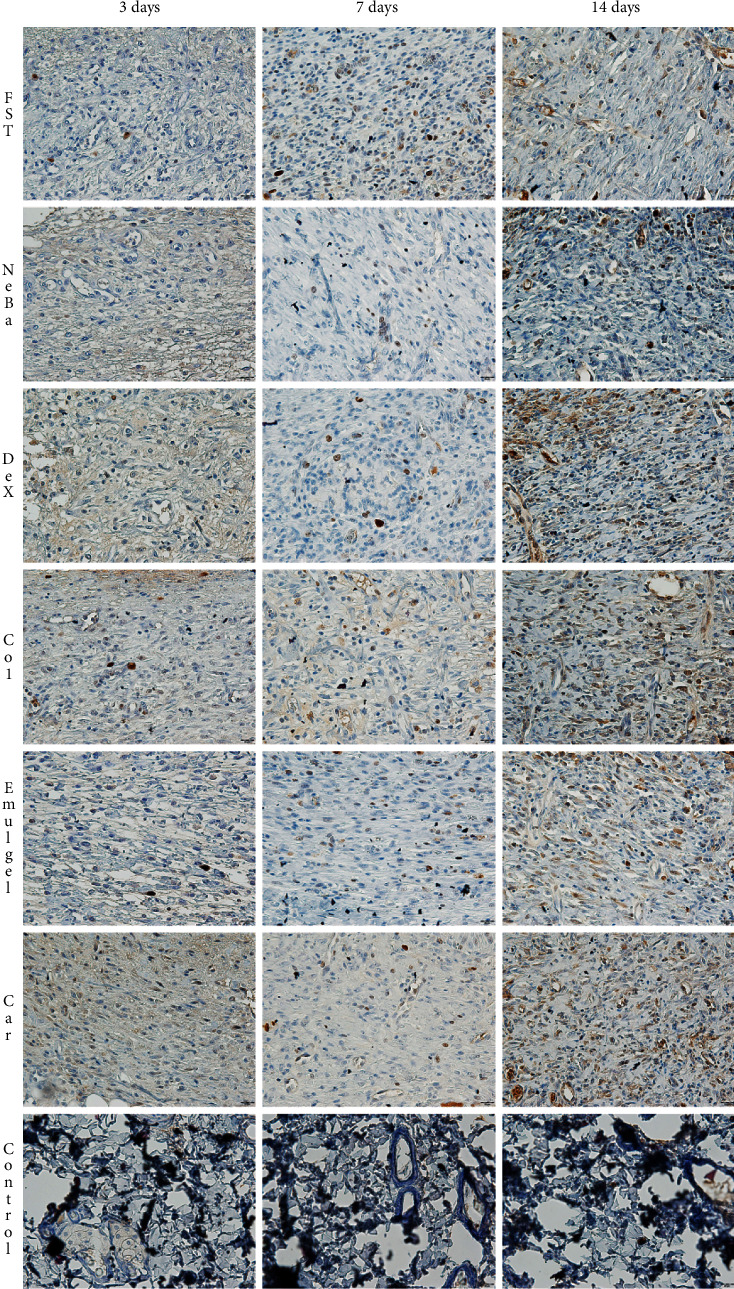
Photomicrographs of the immunolabeling of Ki-67 of the center of the wounds in the dermis of FST, NeBa, Dex, Col, Emulgel, Car and Control groups during 3, 7 and 14 days.

There was an increase in *α*-SMA-immunolabeled fibroblasts in the NeBa, Dex, Col and Car samples compared to FST and emulgel samples after three and seven days of treatment with no differences on day fourteen. However, the commercial treatments and Car formulation showed results similar to the control, indicating normalization of *α*-SMA in these groups (Figures 11 and 12). Immunolabeling for Dsg3 showed a reduction in the labeled area of the NeBa and Car groups compared to the FST group after three days. There was a decrease in Dsg3 immunolabeling in the Col and Car groups compared to the FST and Emulgel groups after seven days. There was no significant difference in the last period of treatment (Figures 11 and 13). There was an increase in Lam*γ*2 immunolabeling in the Col and Car groups compared to the FST and Emulgel groups on day three and an increased area in the Car group compared to the FST and Emulgel groups on day fourteen (Figures 11 and 14). There were no significant differences among the wounded groups in the number of Ki-67-immunolabeled cells in the epidermis, border region of central region of the dermis after three, seven and fourteen days (Figures 15–17 and Supplementary materials 3).

## 4. Discussion

*β*-Caryophyllene is a sesquiterpene in many consumable plants and essential oils with related antioxidant, anti-inflammatory and antimicrobial potentials [8–10]. Previous studies using *C. langsdorffii* oil resin as a treatment for skin excision wounds have shown that the best effective concentration of the oil resin was 10%, and the phytochemical profile of the oil resin has been determined [11, 20]. Therefore, we calculated the concentration of the sesquiterpene based on these studies to synthesize the emulgel formulation for this study, resulting in a concentration of 1%. The present study confirmed the wound-healing activity of an emulgel formulation containing 1% *β*-caryophyllene in a rat excision model, demonstrating the antioxidant and anti-inflammatory activities as well as the improvement in remodeling and re-epithelialization mechanisms mediated by the sesquiterpene.

There are some topical drugs on the market with different mechanisms of action to treat wounds aiming to reduce the time of cutaneous healing and treat errors in tissue repair. Topical formulations containing neomycin and bacitracin zinc sulfate are used in the initial phase of skin wounds to prevent injury from infection, one of the major factors of delayed wound healing [5]. Dexpanthenol (5%) is also used in skin injuries with previous studies reporting the proliferative potential of keratinocytes and fibroblasts with this treatment [21]. Another common drug used to treat cutaneous wounds is collagenase, a protease of *Clostridium histolyticum,* which has been show to play a role in the debridement of provisional extracellular matrix and to have a remodeling mechanism that makes it useful to treat wounds in the clinic [22, 23]. Therefore, these three drugs were used in our study as positive controls to compare the new treatment containing *β*-caryophyllene.

AST, ALT and *γ*-GT are enzymes that catalyze reactions in some peptides and are highly expressed in hepatocytes. When drugs cause a toxic effect in the liver, there is an increase in these enzymes in blood flow, and they are used in the evaluation of hepatic toxicity [24]. Creatinine and urea are proteins physiologically synthesized by the body and are filtered by the kidneys for elimination from the organisms. The increase in plasma levels of these proteins represents the instability of renal filtration due to a toxic effect [25]. The plasma levels of hepatic and renal proteins of *β*-caryophyllene and positive controls were similar to those measured for physiological quantification (Control), demonstrating that the local treatment containing the sesquiterpene did not have systemic toxicity.

Cutaneous injury results in the synthesis and release of mediators of the hemostatic cascade and the death of cells in the wounded area with the local release of reactive oxygen species (ROS) [26]. Although the released free radicals and ROS have important roles in the antimicrobial pathway, exacerbated release of ROS can cause chronic inflammation and impairment in the healing process, resulting in hypertrophic scars or unhealed wounds [27]. To control the concentration of ROS, the cells of the area synthesize antioxidant mediators that are involved in the superoxide and hydrogen peroxide pathways. Superoxide radicals are highly reactive molecules that are processed into hydrogen peroxide, a less reactive molecule, by SOD [16]. However, at high concentrations, hydrogen peroxide is a toxic compound to cells. Thus, hydrogen peroxide is metabolized by CAT and GPx (with consumption of GSH), resulting in water and oxygen molecules [18, 28]. Previous studies have reported the *in vitro* antioxidant potential of *β*-caryophyllene [8, 29], and our findings showing that GPx increased activity confirmed the antioxidant potential of *β*-caryophyllene in a rat excision wound model as well as in the NeBa and Col positive controls.

The inflammatory mechanism is essential for the correct healing of skin wounds. The IL-1*β*, IL-6 and TNF-*α* pro-inflammatory cytokines are involved in cell differentiation and proliferation, coordinating the synthesis of granulation tissue, angiogenesis, re-epithelialization and collagen remodeling mechanisms [30]. Furthermore, these cytokines are associated with IFN-*γ* to enhance the migration to and proliferation of leukocytes at the wound, improving the debridement of necrotic tissue and the phagocytosis of antigens [31]. IL-10 is another interleukin involved in the inflammatory mechanism of skin wounds, acting as an anti-inflammatory mediator inhibiting the synthesis of pro-inflammatory cytokines and playing a role in angiogenesis [32]. However, the imbalance among inflammatory cytokines can lead to a chronic inflammation process, resulting in errors in the subsequent healing mechanisms and the impairment of wound healing [33]. In the present study, the cytokine quantification data showed that the anti-inflammatory activity of *β*-caryophyllene in cutaneous wounds was due to the reduction in IFN-*γ*, IL-1*β*, IL-6 and TNF-*α* levels as well as the increase in IL-10 level with anti-inflammatory potential similar to NeBa, Dex and Col.

Another mechanism of wound healing involves the contraction of wounds with the interaction of fibroblasts and extracellular matrix proteins resulting in wound closure [34]. TGF-*β*1 is a growth factor involved in mechanisms in all phases of wound healing, including the differentiation of fibroblasts. Through TGF-*β*1 stimulation, fibroblasts located at the border of injuries synthesize *α*-SMA, an intracellular stress fiber protein, acquiring a contractile phenotype and differentiating into myofibroblasts [35]. The myofibroblasts bind to the collagen I extracellular fibers and initiate the contraction of the stress fibers, reducing the wounded area. [35]. Thus, the role of the *β*-caryophyllene formulation in wound contraction was validated through macroscopic evaluation, in which a decrease in wounded area was observed in groups treated with *β*-caryophyllene, neomycin/bacitracin and collagenase. Moreover, the immunohistochemical data of *α*-SMA suggested that the increase of wound contraction of these groups was mediated by *α*-SMA as indicated by the increase of immunolabeling of this protein in these treatments.

Re-epithelialization is important in cutaneous wound healing with the proliferation and migration of keratinocytes from the border of injuries. First, proteinases dissolve adhesion molecules among keratinocytes, such as desmoglein-3. These cells proliferate and synthesize the anchoring protein, laminin-*γ*2, to assist in keratinocyte migration through the extracellular matrix, coordinating the re-epithelialization mechanism [36, 37]. However, the overexpression of proliferating mediators causes the hyperproliferation of keratinocytes at the wounds with an increase of epidermis thickness as a marker of keloid formation [38]. Therefore, the histological results of epidermis thickness and the immunohistochemical data of keratinocyte proliferation (Ki-67), desmoglein-3 and laminin-*γ*2 demonstrated that *β*-caryophyllene and collagenase treatments enhanced the re-epithelialization mechanism in which desmoglein-3 and laminin-*γ*2 involved during the migration of cells. Our findings corroborated the results of Koyama et al. [39], who reported enhanced re-epithelialization of skin wounds after treatment with *β*-caryophyllene oil in mice [39].

Finally, the provisional extracellular matrix is replaced by permanent tissue to initiate the remodeling mechanism. In this process, type III collagen and other components of the provisional matrix are metabolized by extracellular matrix metalloproteinases. Simultaneously, compounds, such as type I collagen, elastin and proteoglycans, are synthesized and organized as more resistant tissues, and excess myofibroblasts, fibroblasts and endothelial cells proliferate during healing and undergo apoptosis [40]. Our histological evaluation results of Masson's trichrome staining suggested the role of *β*-caryophyllene in skin repair, increasing collagen synthesis in the central area of wounds during the first period of cutaneous healing with better results in the remodeling mechanism compared to the reference drugs.

## 5. Conclusions

Considering our results, we conclude that the emulgel formulation containing 1% *β*-caryophyllene enhances *in vivo* skin wound healing through antioxidant, anti-inflammatory, wound contraction, re-epithelialization and remodeling mechanisms. Our results demonstrated the potential of *β*-caryophyllene in skin wound therapy compared with three reference drugs, with better results in wound healing compared to reference drugs. Furthermore, the analysis of hepatic and renal parameters confirmed the safety of the tested formulation by showing that there was no systemic toxicity. Therefore, this study provides good evidence that 1% *β*-caryophyllene has great potential for use in treating full-thickness skin wounds, demonstrating the safety and effectiveness of this drug as a future alternative treatment for wound healing.

## Figures and Tables

**Table 1:** Systemic toxicity analysis data for liver (AST, ALT, *γ*-GT) and renal (creatinine, urea) parameters in the serum of rats treated for 14 days.

Groups	AST (IU/L)	ALT (IU/L)	*γ*-GT (IU/L)	Creatinine (mg/dL)	Urea (mg/dL)
FST	143 ± 28	62 ± 12	1.2 ± 0.4	0.30 ± 0.03	44 ± 2.1
NeBa	138 ± 17	65 ± 7.7	1.1 ± 0.3	0.31 ± 0.04	44 ± 6.2
Dex	150 ± 28	80 ± 17	0.9 ± 0.3	0.27 ± 0.03	43 ± 5.6
Col	153 ± 29	63 ± 12	1.0 ± 0.2	0.30 ± 0.05	45 ± 4.6
Emulgel	146 ± 6.1	65 ± 8.5	1.1 ± 0.2	0.26 ± 0.04	44 ± 4.7
Car	124 ± 14	60 ± 9.5	1.1 ± 0.2	0.31 ± 0.03	42 ± 7.6
Control	147 ±25	65 ± 9.1	1.0 ± 0.2	0.30 ± 0.02	44 ± 4.9

One-way ANOVA followed by Tukey test, with p ≤0.05 (n =5). FST: wounded animal without treatment; NeBa: wounded animal treated with neomycin 5 mg/g + sulfate bacitracin zinc 250 IU/g; Dex: wounded animals treated with dexpanthenol 5%; Col: wounded animals treated with collagenase 1.2 IU; Emulgel: wounded animals treated with emulgel (vehicle); Car: wounded animals treated with *β*-caryophyllene emulsion; Control: animals without lesion and treatment – physiologic pattern.

## Data Availability

The informations used to support the findings of this study are included within the article.

## Abbreviations

*α*-SMA: *α*-Smooth muscle actin
*γ*-GT: *γ*-Glutamyl transferase
ALT: Alanine aminotransferase
ANOVA: Analysis of variance
AST: Aspartate aminotransferase
CAT: Catalase
Dsg3: Desmoglein-3
ELISA: Enzyme-linked immunosorbent assay
GPx: Glutathione peroxidase
GSH: Reduced glutathione
HE: Hematoxylin and eosin
HRP/DAB: Horseradish peroxidase/diaminobenzidine
IBB: Institute of Biosciences of Botucatu
IFN-*γ*: Interferon-*γ*
IL-10: Interleukin-10
IL-1*β*: Interleukin-1*β*
IL-6: Interleukin-6
IU: International units
Lam*γ*2: Laminin-*γ*2
MMP: Extracellular matrix metaloproteinases
ROS: Reactive oxygen species
SOD: Superoxide dismutase
TNF-*α*: Tumor necrosis factor-*α*
UK: United Kingdom
UNESP: São Paulo State University
USA: United States of America.
