# Supplementary material for: Beta-caryophyllene as an antioxidant, anti-inflammatory and re-epithelialization activities in a rat skin wound excision model
Source: Oxid Med Cell Longev. 2022 Feb 3;2022:9004014. doi: 10.1155/2022/9004014 (PMC8831077; doi:10.1155/2022/9004014)
Supplement: Supplementary Materials — Supplementary data associated with this article can be found, in the online version of the paper. [file 9004014.f1.docx]

**Supplementary Materials**


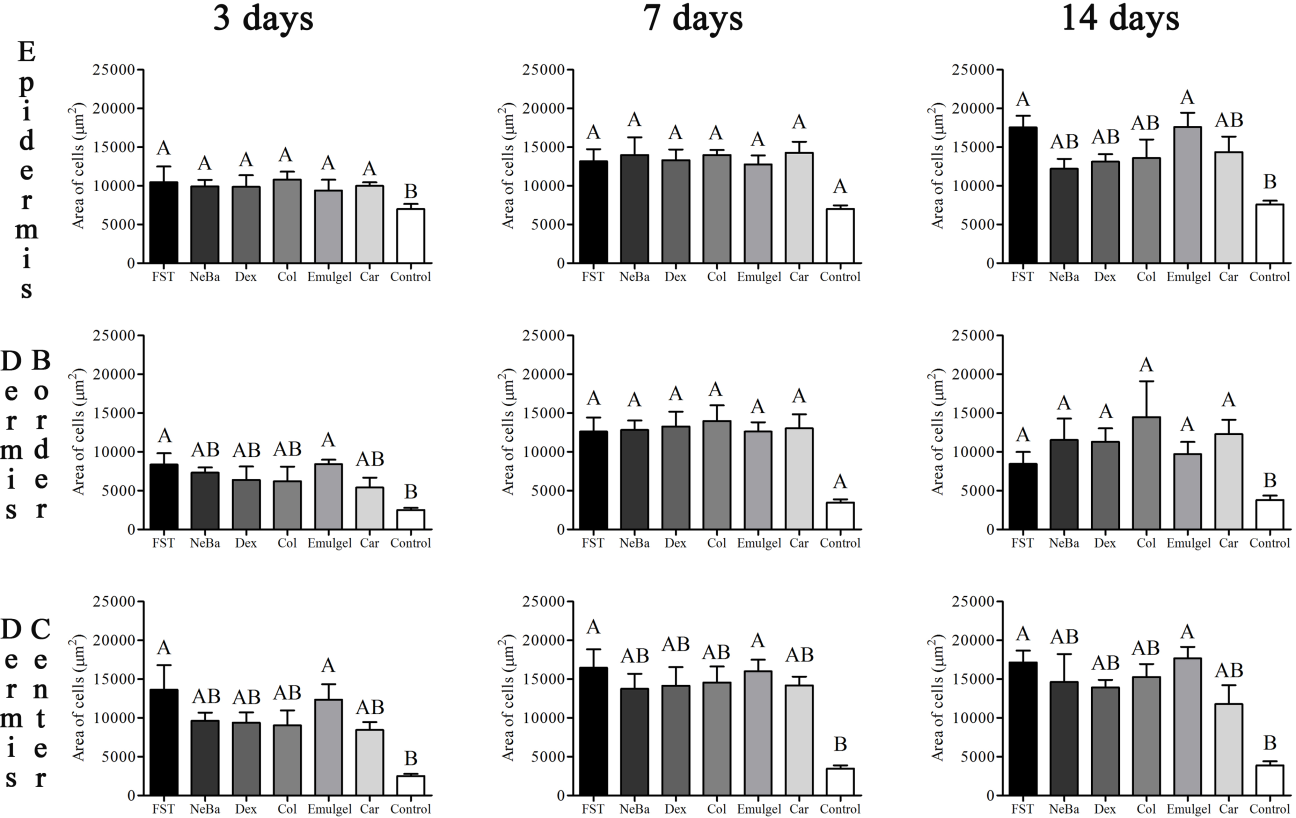


**Supplementary materials 1.** Quantification of cells (µm^2^) in the epidermis, border and center of the dermis in wounds of FST, NeBa, Dex, Col, Emulgel, Car and Control groups during 3, 7 and 14 days. Equal letters show no statistical difference and different letters indicate statistical difference compared to the other groups, according to the Kruskal-Wallis test, followed by Dunn post-test (n = 5).


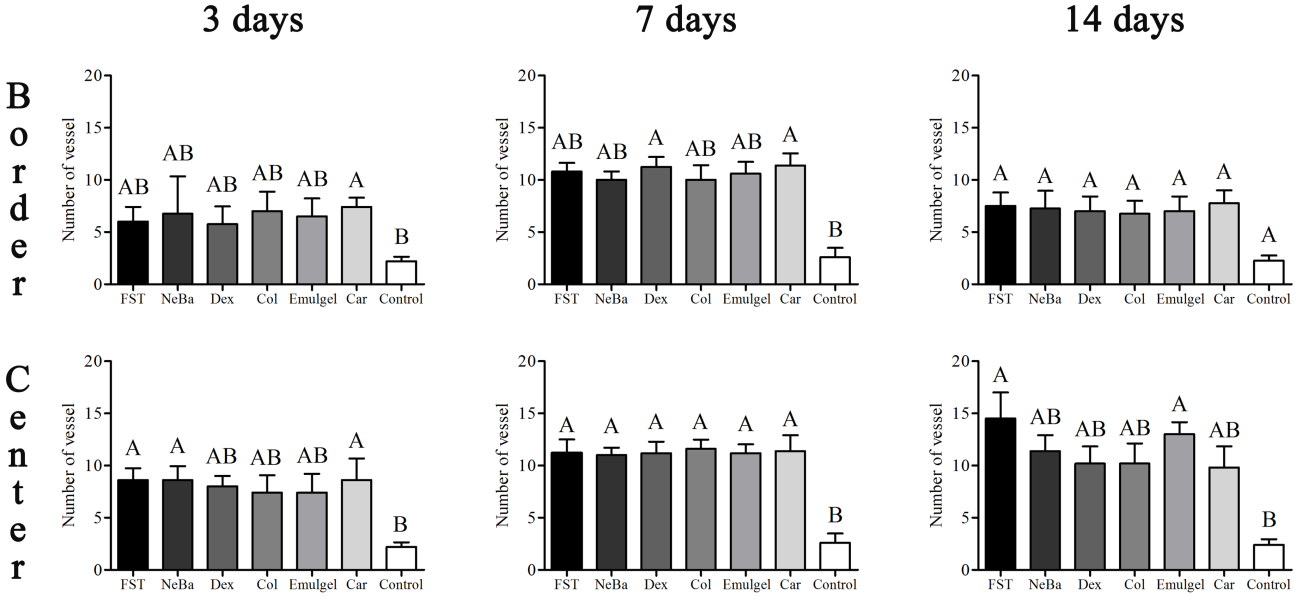


**Supplementary materials 2.** Number of blood vessels in the border and center of the dermis in wounds of FST, NeBa, Dex, Col, Emulgel, Car and Control groups during 3, 7 and 14 days. Equal letters show no statistical difference and different letters indicate statistical difference compared to the other groups, according to the Kruskal-Wallis test, followed by the Dunn post-test (n = 5).


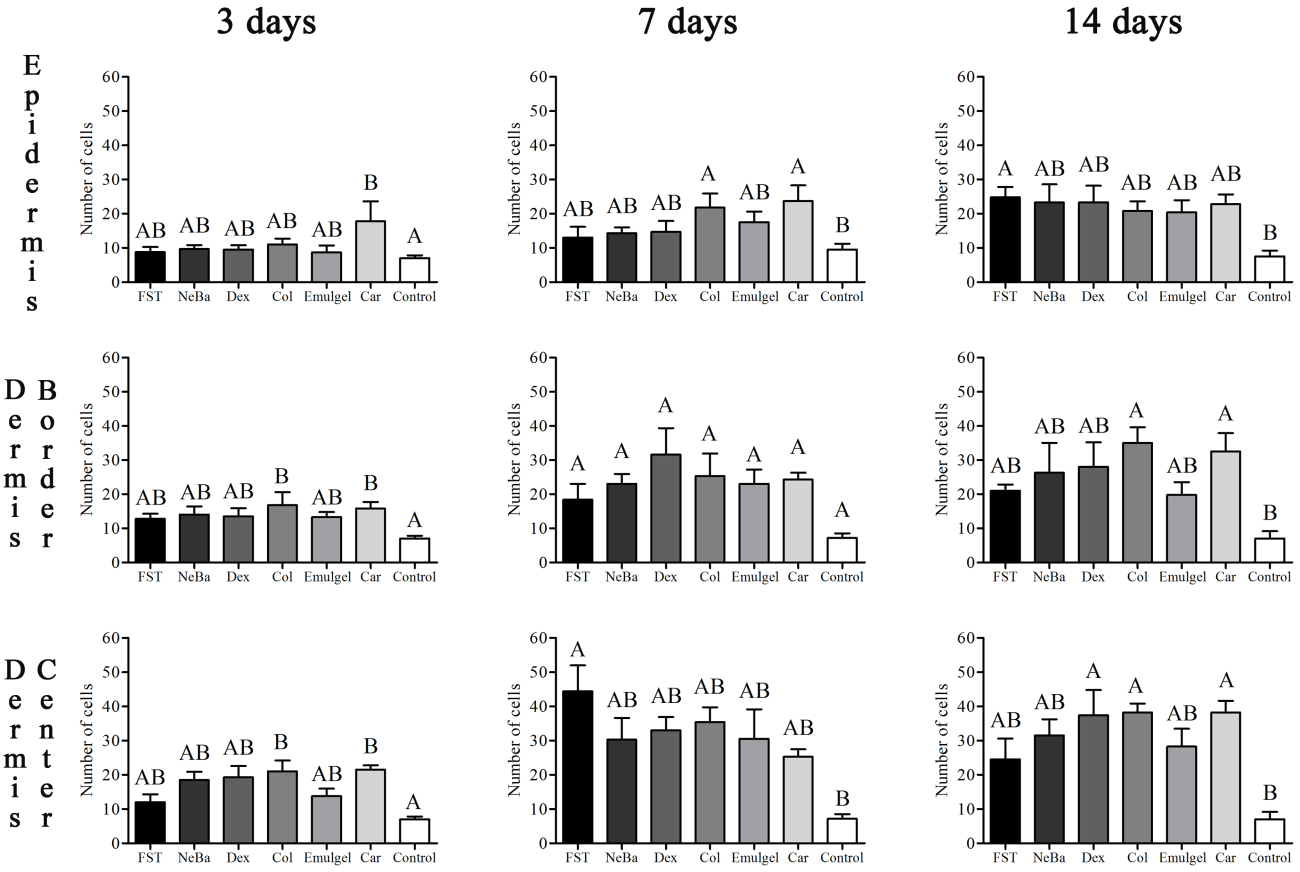


**Supplementary materials 3.** Ki-67 immunolabeling of proliferating cells in the epidermis, border and center of the dermis in FST, NeBa, Dex, Col, Emulgel, Car and Control groups during 3, 7 and 14 days. Equal letters do not show statistical difference and different letters indicate statistical difference compared to the other groups, according to the Kruskal-Wallis test, followed by the Dunn post-test (n = 5).
